# Patients’ experiences of participating in physiotherapeutic telerehabilitation for postpartum pelvic floor dysfunction; a qualitative study

**DOI:** 10.1186/s12891-026-10474-z

**Published:** 2026-09-11

**Authors:** Helena Jönsson, Mari Lundberg, Annelie Gutke

**Affiliations:** 1 Department of Health and Rehabilitation, Unit of Physiotherapy, Institute of Neuroscience and Physiology, Sahlgrenska Academy, University of Gothenburg, Gothenburg, Sweden; 2 Department of Health Promoting Science, Sophiahemmet University, Stockholm, Sweden; 3 University of Gothenburg Centre for Person-Centered Care (GPCC), Sahlgrenska Academy, University of Gothenburg, Gothenburg, Sweden; 4 Pain in Motion (PAIN) Research Group, Department of Physiotherapy, Human Physiology and Anatomy, Faculty of Physical Education & Physiotherapy, Vrije Universiteit Brussel, Brussels, Belgium; 5 Department of Health and Rehabilitation, Unit of Physiotherapy, Institute of Neuroscience and Physiology, Sahlgrenska Academy, University of Gothenburg, Gothenburg, Sweden; 6 Women in Motion Research Group, Department of Health and Rehabilitation, Unit of Physiotherapy, Institute of Neuroscience and Physiology, Sahlgrenska Academy, University of Gothenburg, Gothenburg, Sweden

**Keywords:** Pelvic floor, Women´s health, Physical therapy, Telerehabilitation, Digital care, Content analysis

## Abstract

**Background:**

Postpartum pelvic floor dysfunction is a common condition, yet access to evidence-based rehabilitation remains limited. Access to telerehabilitation is growing, but there is a lack of knowledge about patients’ experiences of participating in telerehabilitation. The aim of this study was to explore patients’ experiences of participating in physiotherapeutic telerehabilitation for postpartum pelvic floor dysfunction.

**Methods:**

Women who had participated in postpartum pelvic floor telerehabilitation were recruited by physical therapists in a primary health care setting across Sweden. Individual semi-structured interviews were conducted with 14 participants. The interviews were transcribed verbatim and analyzed using qualitative content analysis with an inductive approach.

**Results:**

The findings generated the main category, “Telerehabilitation – a complementary piece of a puzzle”, with three underlying categories: (1) *Digital format enables equity*, highlighting improved accessibility, flexibility, and support for active participation; (2) *Digital participation requires advanced communication*, emphasizing the importance of communicating skills, body awareness, and reliable technology; and (3) *Doubting digital format*, reflecting concerns regarding the absence of physical examination, hands-on guidance, and interpersonal interaction.

**Conclusions:**

In this study, telerehabilitation was perceived as an appropriate complement to physical visits for patients with postpartum pelvic floor dysfunction, improving accessibility and continuity of care. Through implementing a hybrid model of care, it may be possible to offer more person-centered rehabilitation care. These findings provide valuable insights from the patient’s perspective that can guide physical therapists and policy makers in tailoring postpartum pelvic floor rehabilitation interventions.

**Clinical trial number:**

Not applicable.

## Introduction

Giving birth is associated with several health risks. Vaginal delivery may involve physical trauma to the pelvic floor and substantial muscle stretching, making it a significant risk factor for pelvic floor dysfunction [1]. Pregnancy itself is also identified as a risk factor for pelvic floor dysfunction [2]. The pelvic floor plays a crucial role in supporting the pelvic organs, contributing to sexual function, ensuring continence, assisting in voiding and defecation [3], as well as contributing to postural control [4] and proper load transfer through the lumbopelvic region [5]. A well-coordinated interaction between the muscles, ligaments, and fascia, dependent on a well-functioning neuromuscular system, is essential for maintaining pelvic floor function [6]. Pelvic floor dysfunction, characterized by abnormal functioning of these components, can cause conditions such as urinary and anal incontinence, pelvic organ prolapse, difficulty emptying the bladder and bowel, sexual problems, and pain [3], which negatively impact a woman’s well-being and quality of life [7].

Given the heterogeneity of pelvic floor dysfunction, physical therapy management is tailored to the individual’s symptoms and clinical findings. Treatment may include pelvic floor muscle training or relaxation, manual therapy, behavioral and lifestyle interventions, biofeedback, and neuromuscular electrical stimulation [8]. While these interventions are used across a range of pelvic floor disorders, the strongest evidence supports pelvic floor muscle training for urinary incontinence, for which it is recommended as a first-line treatment [9, 10], and for reducing symptoms of pelvic organ prolapse [11]. Comprehensive physical therapy assessment and treatment often require internal palpation of the pelvic floor muscles [12, 13].

Since 2005, the World Health Organization has been urging countries to strategically develop and implement digitalization in healthcare [14]. The COVID-19 pandemic has accelerated this development [15, 16], opening up new opportunities for the treatment of pelvic floor dysfunction [17]. One systematic review and meta-analysis indicates that physiotherapeutic telerehabilitation is a potential contributor to reducing urinary incontinence and improving quality of life [18]. Telerehabilitation is defined as the remote delivery of rehabilitation services using information and communication technologies, including real-time video consultations between patients and physical therapists [19]. Research has predominantly followed a positivistic tradition, leaving a gap in understanding patients’ experiences. In postpartum pelvic floor dysfunction, where symptoms are intimate and rehabilitation requires active patient engagement, a significant knowledge gap remains regarding patients’ experiences of telerehabilitation in practice. Qualitative methods are therefore considered valuable to complement existing knowledge by increasing the understanding of patients’ experiences [20]. By better understanding the patients’ perspective, the health profession can more effectively tailor personalized interventions for the patient.

Therefore, the aim of this study was to explore patients’ experiences of participating in physiotherapeutic telerehabilitation for postpartum pelvic floor dysfunction, specifically rehabilitation via synchronous video consultation with a physical therapist.

## Methods

### Study design

Individual, semi-structured interviews were conducted. The data were analyzed using a qualitative content analysis with an inductive approach [21]. The study is reported according to the Consolidated Criteria for Reporting Qualitative Research (COREQ) checklist [22].

### Qualitative approach and research paradigm

The methodological approach was inductive, and involved searching for patterns, similarities and differences in the collected material [23]. Coded categories were derived directly from the textual data, with the advantage of obtaining information directly from patients [24]. The qualitative content analysis method, which originated from a positivist paradigm rooted in quantitative analysis, incorporates both phenomenological and hermeneutic elements, as texts are described, abstracted, and interpreted at various levels [23]. In qualitative content analysis, ontological assumptions are seen as flexible and dependent on the researcher’s point of view, while epistemologically, both the interviewer and the interviewee are seen as co-creators of data, with interpretation during analysis being a collaborative endeavor between the researcher and the text [23]. The focus was on describing the phenomenon under study by analyzing the manifest content, as reflected in categories and subcategories. The analysis of manifest content involves interpretation, but the depth and level of abstraction varies in relation to the analysis of latent content [25].

### Description of study researchers

To facilitate a broad understanding of the phenomenon under study, the research group consisted of three researchers with different competencies and experiences. Annelie Gutke (AG) is a physical therapist specializing in gynecology, obstetrics, and urology, who has been working clinically with women with pelvic floor dysfunction for 20 years and also has experience in telerehabilitation. Mari Lundberg (ML) is a physical therapist and professor in physical therapy, with experience and skills in conducting qualitative research studies and content analysis. Helena Jönsson (HJ) is a physical therapist with 16 years of clinical experience, specializing in working with patients with pelvic floor dysfunction.

### Participants

Women over 18 years old, who had given birth at least once, and who had participated at least once in a video consultation with a physical therapist related to their pelvic floor dysfunction within the first year postpartum, were asked to participate. They were excluded if they were unable to speak and understand the Swedish language, or if they had received telerehabilitation for pelvic floor dysfunction before childbirth. The study aimed to include 10–15 participants as an initial estimate to ensure variation and data richness [21, 23].

### Inclusion procedure

Study participants were recruited by physical therapists in Sweden who work with pelvic floor rehabilitation. Physical therapists identified through nationwide networks were informed about the study by email, and in turn approached patients through direct requests, participant recruitment advertisement, and social media. The recruiting physical therapists informed HJ about interested patients and provided contact details. HJ then contacted these patients for further details. Written informed consent was obtained before participation. The study was approved by the Swedish Ethical Review Authority.

Participants were selected using strategic sampling based on factors such as age, occupation, education level, place of residence, number of video consultations, cultural background, and symptoms. A total of 19 patients expressed an interest in participation, and after reviewing the criteria, 14 were included.

### Context

In Sweden, postpartum physical therapy is not routinely incorporated into standard postpartum care. Although patients have direct access to physical therapy within primary care without the need for physician referral, access to physical therapists specialized in pelvic floor dysfunction is limited [26]. In this study, participants received rehabilitation within a primary care setting, reflecting the standard level of physical therapy services available in the Swedish healthcare system [27]. In Sweden, the publicly funded parental leave system provides extensive income-related benefits and job protection for parents, including 480 days of paid leave per child that can be shared between caregivers, creating a social context in which pregnancy and early parenthood are strongly supported by the welfare state [28].

### Data collection

Data were collected through individual semi-structured interviews using an interview guide developed by the research group (Appendix 1). HJ conducted ten of the interviews by telephone and four by video call, depending on the participant’s preference, between February 2022 and March 2023. Questions were asked naturally, not in a specific order, and follow-up questions were used to deepen the understanding. After a pilot interview, minor adjustments were made. The pilot interview was included in the analyses. The interviews were audio-recorded and lasted between 25 and 46 min, with an average of 34 min.

### Data processing

All data were pseudonymized using letter codes, and only HJ had access to the key for the codes. HJ transcribed all interviews verbatim, and the text was the unit of analysis. Transcripts were not returned to participants for comment or correction.

### Data analysis

**Fig. 1:**
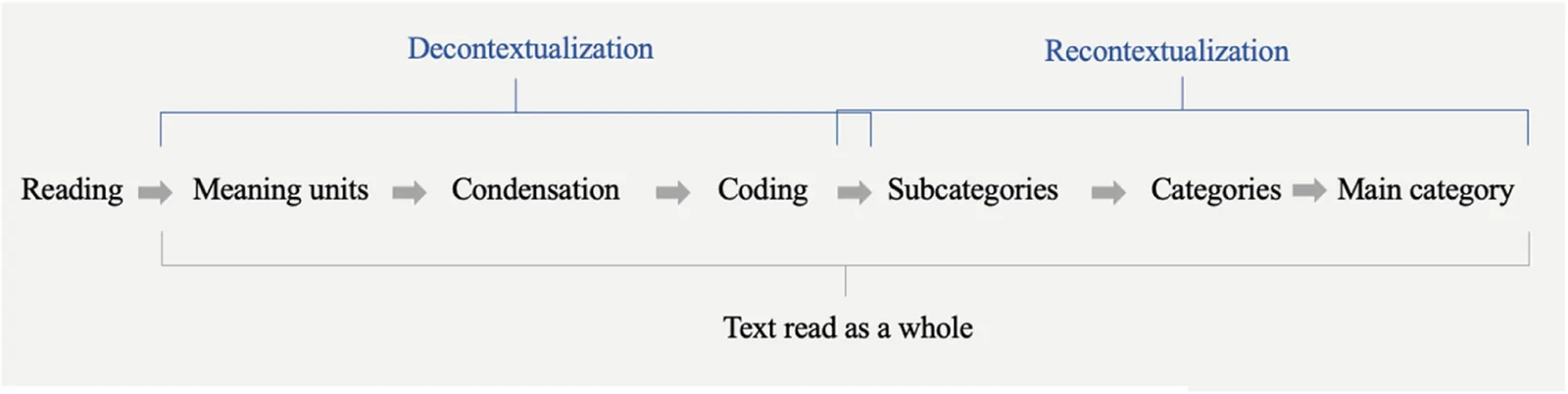
Steps in the analysis of the transcribed interview text

The transcribed text was analyzed using inductive content analysis inspired by Graneheim and Lundman [21]. The focus was on the manifest content, and the steps of the analysis process are illustrated in Fig. 1 and clarified by examples in Table 1. The text was read several times both before and during the analysis to ensure a comprehensive understanding. HJ initiated the analysis, supported by ML, and performed decontextualization by identifying and condensing meaning units without losing their message, which were then coded. AG joined in the recontextualization, where the codes were compared with each other and sorted into subcategories. The subcategories were analyzed and grouped into categories. The comparison, analysis, and interpretation of the subcategories and categories, together with the whole text, led to identification of the main category. The analysis was carried out between March 2023 and October 2024.

**Table 1:** Example to clarify the analysis process

Meaning unit	Condensation	Code	Subcategory	Category	Main category
You kind of think before you need any care that it''s the same care throughout the country,but that''s absolutely not the case. So the digital visits really help withthat, so even if you live somewhere where there is no such competence you can take part in it; it''s better in any case. (I)	Digital visits bring care closer and provide access to expertise across the country	Access to specialist help increases via digital visits	Digital format enables accessible rehabilitation	Digital format enables equity	Telerehabilitation- a complementary piece of a puzzle

### Techniques to enhance trustworthiness and credibility of data analysis

To enhance trustworthiness and credibility of the data analysis, a procedure plan was developed a priori (see Appendix 2). To increase credibility, multiple researchers were involved. ML systematically supervised all steps of the analysis and there was continuous discussion and reflection. Different levels of abstraction and interpretation were applied during the analysis process. In reporting the results, quotations are frequently presented to provide clarity and enhance the credibility of the findings.

## Results

**Fig. 2:**
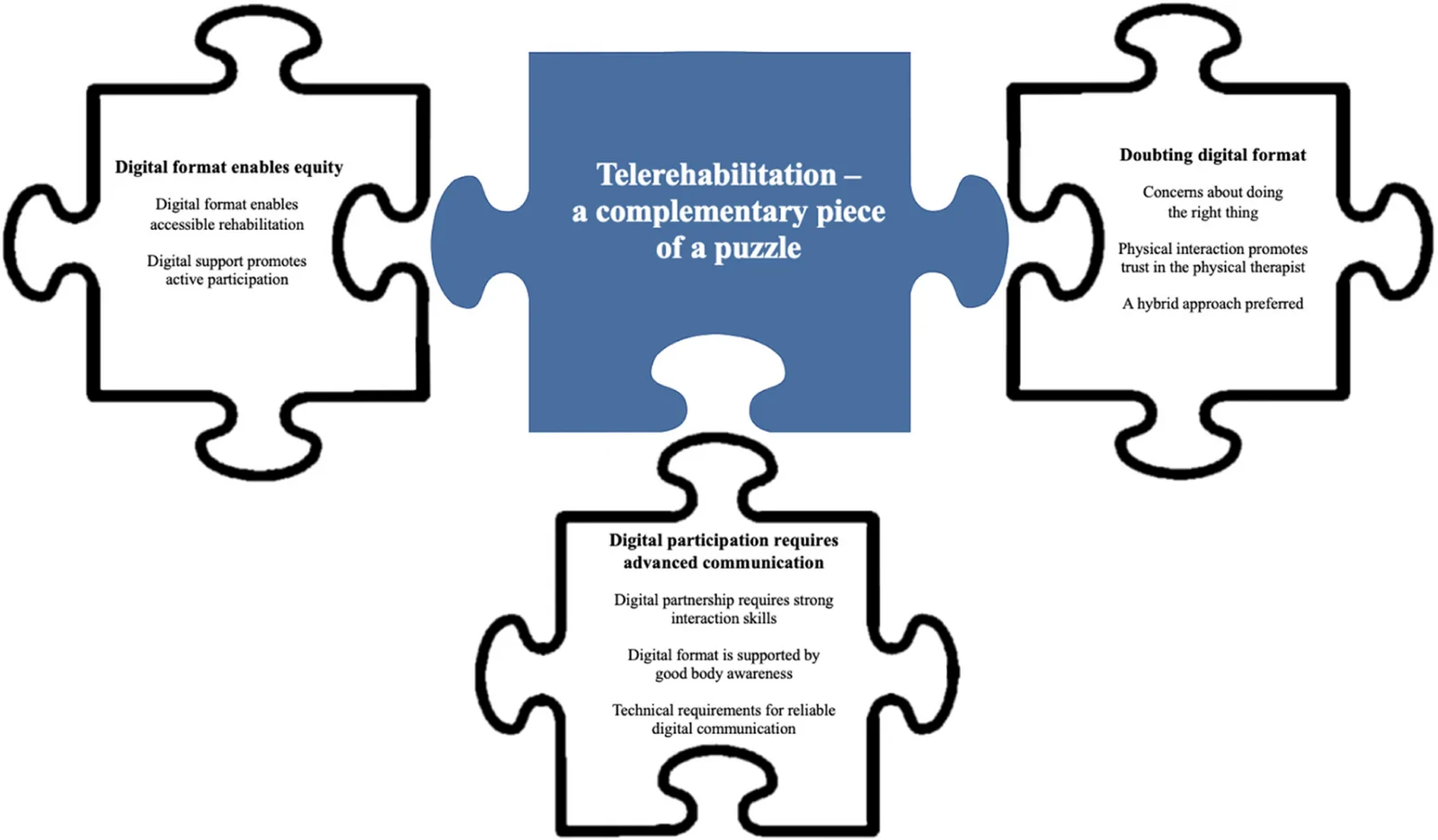
Overview of results showing the main category, categories, and subcategories

This study included 14 women (aged 28–41 years) (Table 2). Interviews were conducted approximately 4–28 months postpartum. The time elapsed between telerehabilitation and the interview varied between participants. The participants’ pelvic floor symptoms varied in type and severity. Five of the participants had video consultations only, while nine had both video consultations and physical visits during the rehabilitation process. The content analysis identified the main category, “Telerehabilitation – a complementary piece of a puzzle,” and three underlying categories, each with two to three subcategories, which together illustrate experiences of participating in physiotherapeutic telerehabilitation for postpartum pelvic floor dysfunction (Fig. 2).

**Table 2:** Participants'' characteristics

Number of participants	14
Age (yrs)	
Mean (SD)	33 (3)
Median	33
Range min-max	28-41
Country of birth	
Sweden	11 (79%)
Germany	1 (7%)
Colombia	1 (7%)
Canada	1 (7%)
Place of residence	
City	8
Urban area	4
Small town	2
Level of education	
High school	1 (7%)
College	13 (93%)
Occupation	
Employed	13 (93%)
Student	1 (7%)
Number of children	
One child	8 (57%)
Two children	5 (36%)
Three children	1 (7%)
Mode of delivery	
Vaginal	12 (86%)
Vaginal and Cesarean	2 (14%)
Number of video consultations	
Mean (SD)	3 (3.75)
Median	2
Range min-max	1-15
Also had at least one physical visit to the physical therapist	
Yes	9 (64%)
No	5 (36%)

### Main category: “Telerehabilitation – a complementary piece of a puzzle”

As reflected in the narratives, the participants saw telerehabilitation as a complementary piece of the rehabilitation puzzle. On the one hand, they expressed that telerehabilitation can facilitate women to participate in pelvic floor rehabilitation regardless of distance, the family situation, or a reluctance to attend physical visits. The digital format can support rehabilitation by enabling continuous access to communication, guidance, and follow-up with the physical therapist, alongside support from family members. On the other hand, the participants had doubts about the trustworthiness of telerehabilitation in the absence of physical interpersonal interaction and a physical examination. Telerehabilitation, in contrast to attendance at treatment appointments, relies on well-functioning technology and advanced communication skills of both the patient and the physical therapist.

#### Category: digital format enables equity

The digital format supported equity in pelvic floor rehabilitation by addressing patients’ individual needs and accommodating different life circumstances. Women who might otherwise not have sought care because of the intimate nature of their symptoms, feelings of shame, or the practical challenges of caring for a newborn were offered a model of care that better accommodated their individual circumstances. In this sense, the digital format supported more equitable participation in pelvic floor rehabilitation by creating opportunities for women facing different barriers and life circumstances to engage in rehabilitation. This was further supported by enhanced accessibility, faster access to care, and broader access to specialized physical therapy. Digital access also enabled continuous participation and support throughout the rehabilitation process, as patients could attend sessions from home, including when caring for a sick child. This flexibility was perceived as a factor that helped patients remain active and engaged in rehabilitation. The digital format was considered superior to the physical format in terms of flexibility since it was perceived as less resource-intensive in terms of time and energy.

##### Subcategory: digital format enables accessible rehabilitation

The participants described how digital access to physical therapy enabled rehabilitation that would not otherwise have been possible. They expressed that the digital format provided a more accessible avenue to receive care, which was particularly beneficial for individuals who were reluctant to seek physical care. The remoteness of the digital format could create a sense of security, reduce embarrassment, and facilitate open communication about pelvic floor problems. The ability to avoid a physical examination could be a motivating factor for choosing video consultations, making it easier to seek care. The option to get care remotely provided faster access to care and specialist help regardless of location. Moreover, the digital format was seen as enabling rehabilitation for patients with physical impairments who would otherwise have difficulty getting to the clinic.

*I really would not have had the quality of life that I have today if I had not had these visits. And I would have found it harder to attend physical appointments when I was at my worst. So it was very good that I could do it from home and that I didn’t have to travel to Gothenburg because that is quite a distance. I wouldn’t have been able to do that*,* not physically. (D)*

##### Subcategory: digital support promotes active participation

The participants described how the telerehabilitation facilitated continuous support from the physical therapist throughout the rehabilitation process, despite barriers to in-person care such as caregiving responsibilities or illness affecting either the patient or the physical therapist.

The presence of support was perceived as a motivator to perform home exercises more often. Video consultation also made it easier for family members to attend, enabling them to be involved and support the rehabilitation process.

*To do the pelvic floor exercises every day*,* several times a day*,* is quite*,* well*,* it’s difficult. It’s really hard to motivate yourself*,* so I think it’s very important to get that support and motivation from time to time*,* like*,* for example*,* reassurance that things are going in the right direction and confirmation that*,* yeah*,* it’s important to do them and so on. Because it’s quite*,* yeah*,* it’s quite boring to do the exercises and it takes a long time to get better*,* so you don’t see a quick improvement*,* which can be a little hard sometimes. (K)*

#### Category: digital participation requires advanced communication

Building a partnership in the digital format was seen as requiring strong interpersonal and communication skills from both the physical therapist and the patient, as the communication had to be especially responsive and clear in the absence of physical interaction and demonstration. The patient’s body awareness was considered a crucial factor for participating in rehabilitation in the digital format. Well-functioning technology was seen as an essential prerequisite for digital participation, as was technical competence on the part of both the physical therapist and the patient.

##### Subcategory: digital partnership requires strong interaction skills

Participants emphasized that the physical therapist’s competencies in being present and responsive were essential to making them feel seen in the digital meeting. The presence of a responsive physical therapist was found to encourage openness in the digital partnership. Since there was no room for physical interaction and demonstration, the physical therapist’s communication skills and pedagogical skills were described as crucial in remotely creating understanding and making participants feel understood. The patient’s own ability to ensure that the physical therapist understood their needs was also seen as a crucial cornerstone for creating concordance in the digital meeting.

*I don’t find that it’s difficult to open up about pelvic floor problems to a healthcare provider*,* whether it’s digitally or in person. As long as the person is*,* yes*,* professional and inviting*,* as most of them are. But that communication*,* the nonverbal communication of being open and professional*,* it’s a little more difficult digitally. So if you don’t know*,* if you haven’t sort of met the person before*,* it can be more difficult to get that*,* I don’t know*,* invitation to sort of tell. (C)*

##### Subcategory: digital format is supported by good body awareness

The digital format was considered challenging since it relied solely on the participants’ ability to describe bodily experiences during assessment and rehabilitation. The lack of physical instructions for how to perform pelvic floor exercises made it difficult to understand digital instructions. Prior knowledge, such as a good body awareness, and prior familiarity with the pelvic floor and exercises was deemed crucial as having this knowledge and familiarity gave the participants confidence when receiving telerehabilitation.

*I*,* I think that the process has become more dependent on my own experience more than anything else*,* more than the physical therapist’s perception of my body. That I have needed*,* it’s probably something that could have been different if it was more physical. But now it has needed to be the foundation of it all. (N)*

##### Subcategory: technical requirements for reliable digital communication

Successful telerehabilitation are dependent on reliable technology, with good connectivity, and audio and visual quality as fundamental components. Based on the participants’ narratives, the ability to access a secure, patient-safe platform that ensures privacy and confidentiality was considered reassuring. Lack of clear instructions for video consultations and login procedures could create uncertainty. Technical problems were seen as barriers to the rehabilitation process by hindering communication and understanding. A participant’s technical expertise, including familiarity with video consultations, was considered to create trust in digital communication. In addition, the physical therapist’s ability to assist with technical difficulties was mentioned as important.

*For me*,* it was rather that my sound was a little bit bad. Yeah*,* but there was a little bit that you had to*,* that I had to repeat myself a little bit*,* had to be a little bit too clear and then you don’t talk as much in the end because you want to get what you’re saying out and then you say it a little bit shorter. Yes*,* but it turned out that you don’t really understand*,* yes*,* that you don’t get the whole picture of how it is. (K)*

#### Category: doubting digital format

Participants’ narratives conveyed concerns about relying solely on telerehabilitation in pelvic floor rehabilitation. The ability to contract pelvic floor muscles correctly was considered crucial for successful rehabilitation. The lack of a physical examination, physical guidance, and personal interaction with the physical therapists caused feelings of helplessness and a lack of trust in the rehabilitation process. There was a wish for physical visits in addition to the digital format, to provide reassurance and optimize rehabilitation.

##### Subcategory: concerns about doing the right thing

The participants expressed concerns regarding the reliability of pelvic floor assessment in a telerehabilitation setting. They felt insecure about the accuracy of the remote assessment of their muscle damage and the healing process and were concerned about getting a clear diagnosis without undergoing a physical examination of the pelvic floor. They expressed that the lack of physical guidance in the digital format led to uncertainty about the accuracy of their pelvic floor contraction technique and whether they were performing the rehabilitation exercises correctly. These uncertainties were seen as potentially undermining trust in the rehabilitation process.

*So the most important part*,* you can’t really do digitally because the most important part is*,* well*,* it’s the technique. Yeah*,* but now you’re squeezing much easier compared to the first time or you’re only squeezing with one side or*,* I don’t know*,* can you try pulling a bit more here so you can try reloading and such. That’s something you can only do when the physical therapist has the opportunity to feel the pelvic floor. And you can only do that in person. So with pelvic floor exercises*,* then maybe they might*,* then maybe you’ll get a 3 out of 10 out of it. (C)*

##### Subcategory: physical interaction promotes trust in the physical therapist

Participants who had participated in a physical visit found it easier to establish a trust in the physical therapist. The physical presence fostered a sense of security, and the face-to-face contact encouraged the participants to feel acknowledged and comfortable opening up to the physical therapist. The nonverbal communication in the physical meeting was seen as helping the physical therapist get to know the individual and understand their needs. The physical interaction also facilitated participants’ trust in the physical therapist’s assessment, which provided reassurance that they had been personally observed.

*And I think that maybe I wouldn’t have had the same confidence in her because when I met her she was so thorough and so. Well*,* of course*,* you can be that way remotely too*,* but she really saw all of me when I was there. And that*,* that she knew how I was physically. It gave me confidence that she knows what she’s doing*,* she’s seen me. (D)*

##### Subcategory: a hybrid approach preferred

Video consultations were considered appropriate for initial consultations, information, and follow-up that did not require a physical examination or manual treatment. The participants emphasized the challenges of making progress without the physical therapist being physically present, particularly with certain hands-on interventions such as pelvic floor assessment and manual treatment that could not be delivered in a digital format. In addition, the inability remotely to fully demonstrate physical exercises was cited as a reason for wanting physical visits. The availability of a physical visit itself provided a sense of security in the digital format.

*It could mean that this is very good and empowering for me right now. But if*,* if all the conditions were there*,* if I could have chosen to meet physically as well*,* if I were completely free to choose*,* if there were no obstacles*,* then I would have chosen to meet partly physically and partly digitally. This means that there is security in the physical meeting*,* since this is a physical suffering*,* to be a physical person of flesh and blood in front of another*,* if you need to get an objective assessment and that someone examines the body purely physically. (N).*

## Discussion

The aim of this study was to explore patients’ experiences of participating in telerehabilitation for postpartum pelvic floor dysfunction. The study resulted in the main category; “Telerehabilitation - a complementary piece of a puzzle.” The results and their clinical relevance are discussed below.

Access to evidence-based postpartum pelvic floor rehabilitation may be inconsistent across healthcare settings [29]. The present study suggests that telerehabilitation may improve accessibility and contribute to more equitable care. In this context, equity refers not simply to whether care is available, but to whether care can be provided in ways that acknowledge and accommodate differences in individuals’ circumstances, resources, and barriers rather than providing everyone with the same mode of care. Telerehabilitation may therefore provide an additional means of addressing both individual and structural barriers, helping to ensure that every woman has a fair opportunity to participate in appropriate rehabilitation.

By overcoming barriers such as distance and physical limitations, the remote rehabilitation care made participants feel closer to specialist care. This is consistent with existing research showing that video consultations improve access to care and quality of life for those with limited access [30]. Participants in our study described how, for them, the digital format facilitated the puzzle of daily life and incorporating pelvic floor rehabilitation because it required fewer resources in terms of time and energy, making it easier to balance rehabilitation with parenting and work. Similarly, video consultations in a primary care setting have been seen as valuable because of their convenience, especially for individuals juggling work, study, and child care [31].

Seeking care for pelvic floor dysfunction can be challenging because it is an intimate issue that women may be embarrassed to discuss [32]. Our study indicates that the digital format may lower this threshold, encouraging reluctant individuals to seek care. However, the subjective nature of perceptions of privacy in digital care should be highlighted, as this can both facilitate and hinder care seeking [33]. Some patients have been reported to prefer physical visits during emotionally charged consultations [31]. In the context of pelvic floor dysfunction, younger women and women with a college degree have been found to be more willing than their older, less educated counterparts to engage in digital communication with healthcare providers [34]. This is important to note, as expanding digital care clinically may risk excluding individuals outside of these demographics [35]. A person-centered approach in healthcare should focus on the person’s life situation and tailor interventions based on their emotions, preferences, and resources [36]. In line with this, the results of the present study suggest that the availability of different care formats, including digital formats, is beneficial to accommodate diverse patient preferences and needs.

The results of our study emphasize that telerehabilitation requires advanced communication skills and technical prerequisites on the part of both the patient and the physical therapist, which is supported by others [37]. The participants described that it was more challenging to be seen and feel understood in the digital format. Without strong interpersonal and communication skills, building a therapeutic partnership was perceived as difficult. Communication is seen as the basis for building alliances [38], and adapted communication is seen as critical for establishing a successful therapeutic alliance in telerehabilitation [39]. As the physical aspect is lacking in digital communication, there is a need for physical therapists to adapt their communication style, possibly through training [40, 41], to develop safe digital public health interventions [41]. Consistent with another study [42], the participants in our study felt that digital care worked better when the healthcare provider and the patient already knew and trusted each other. Our findings show that trust in the physical therapist was enhanced by a physical visit. We will argue that this should be considered in the clinical setting when implementing digital care, as trust in the physical therapist is seen as an important factor in patient engagement in rehabilitation [43].

Consistent with previous research [44], suggesting favorable effects on attendance and adherence in telerehabilitation, participants in our study perceived that the digital format facilitated access to physical therapy sessions and provided continuous support despite barriers to in-person care, thereby promoting active engagement in their rehabilitation. This is seen as valuable, as adherence plays a crucial role in achieving outcomes and alleviating symptoms in pelvic floor rehabilitation [45]. However, it remains unclear how physical therapists can effectively deliver telerehabilitation for pelvic floor dysfunction without the ability to physically palpate the pelvic floor muscles to assess function and provide accurate muscle contraction instructions [46]. Evidence has shown that a substantial proportion of women are unable to correctly perform a pelvic floor muscle contraction without physical instruction or feedback, highlighting the importance of guidance during rehabilitation [13, 47]. Consequently, telerehabilitation as the sole mode of care, without the possibility of physical visits, may therefore carry a risk of incorrect pelvic floor muscle contractions and reinforcement of inappropriate movement patterns, such as repeated Valsalva maneuvers or straining, which could potentially exacerbate symptoms in some individuals. This may limit the effectiveness of rehabilitation and prevent patients from achieving optimal rehabilitation outcomes. Similarly, in cases with pelvic floor muscle dysfunction, adjunctive interventions such as electrical stimulation or biofeedback may be required to facilitate correct activation and ensure effective treatment delivery [9], which cannot be provided through telerehabilitation alone. This highlights an important consideration when evaluating the feasibility and safety of telerehabilitation and emphasizes the need to identify which components of pelvic floor rehabilitation can be effectively delivered digitally and when physical care is required. Our results indicate that telerehabilitation requires good body awareness on the part of the patient, and that prior familiarity with pelvic floor exercises is beneficial. The participants raised concerns about their pelvic floor dysfunction and the effectiveness of rehabilitation without a physical examination, echoing findings from other studies [16, 48]. Based on our participants’ narratives, we found that some aspects of pelvic floor rehabilitation, such as the initial consultation and follow-up, where palpation or manual treatment may not be essential, can be effectively delivered digitally.

Our results suggest that a hybrid approach which combines access to both telerehabilitation and physical care is preferred in pelvic floor rehabilitation, in agreement with other research [49]. We therefore argue that telerehabilitation should not replace the possibility of physical visits. This aligns with another study [50] where digital visits for musculoskeletal pain were not seen as a substitute, but rather as a complement to, physical visits.

### Limitations

Regarding dependability, it is important that all participants are asked similar questions. At the same time it is natural for interviews to evolve as the interviewer gains new insights along the way [21]. To increase dependability, an interview guide was developed, but follow-up questions asked during the interviews varied due to their semi-structured format. Interviews were conducted via telephone or video call, which may have the disadvantage of limited nonverbal communication compared with face-to-face interviews [51, 52]. On the other hand, digital interviews offered advantages in terms of accessibility, cost and time [52], particularly facilitating wider geographical participation in this study.

It cannot be excluded that the results would have been different if the research had been conducted by other researchers, as pre-understanding potentially influences the interview and the way narratives are interpreted [21]. However, it is considered an advantage in this study that several researchers with different pre-understandings participated, as this may increase credibility [23]. Transcripts were not returned to participants, nor were they asked to comment on the results, which may be seen as a limitation. To facilitate the reader’s ability to assess the trustworthiness and credibility of the study, a systematic approach has been taken and information has been shared to provide insight into the methodology and analysis process.

We acknowledge that a time delay between the intervention and the conducting of 

interviews may have introduced limitations related to memory and retrospective construction, as participants may have forgotten details or interpreted experiences differently over time [53]. At the same time, the longer interval may also have enabled participants to reflect on and describe the perceived long-term significance of the intervention. Since qualitative content analysis according to Graneheim and Lundman [21] was applied, the analysis focused not only on factual descriptions of events, but also on the meanings and experiences expressed in the interviews. To strengthen credibility and trustworthiness, the analysis involved repeated readings of the transcripts and continuous discussions among the researchers throughout the analytic process [21, 23].

In this study, the material was deemed sufficiently extensive and varied for a credible result, but it cannot be excluded that more participants would have created greater variation. One limitation is the predominance of highly educated women among the participants; a more diverse sample in terms of educational background might have enriched insights. However, the ethnic diversity of the participants provided valuable variation.

The selection of participants also affects the transferability of the results [23]. The results of this study are considered to be transferable to other postpartum pelvic floor dysfunction rehabilitation contexts. Detailed inclusion criteria and participant descriptions are provided to assist the reader in assessing transferability.

## Conclusion

In this study, telerehabilitation was perceived as an appropriate complement to physical visits for patients with postpartum pelvic floor dysfunction, contributing to improved accessibility and continuity. Through implementing a hybrid model of care, it may be possible to offer more person-centered rehabilitation care.

## Data Availability

The datasets used and/or analyzed during the current study are available from the corresponding author on reasonable request.

## Appendix 1

### Interview Guide

- Could you tell me in your own words about what your pelvic floor rehabilitation has involved?
- You’ve had video consultations with a physical therapist regarding your pelvic floor problems. Could you describe to me how the video consultation with the physical therapist went?
- Can you describe in your own words your experience of getting help with your pelvic floor problems from a physical therapist via video consultation?
- What was it like to talk about your pelvic floor problems with the physical therapist in a digital room? (Do you remember how it felt?)
- How did it feel to receive information and instructions from the physical therapist via video consultations? (Could you describe what it was like in practice when you received instructions digitally? Could you elaborate on how it felt?)
- You’ve participated in physiotherapeutic rehabilitation; how has this been affected by the fact that it was done remotely? (Could you elaborate and describe in what ways?)
- What was your experience of the technical aspects of the video consultations?
- In your own words, how would you describe your experience of telerehabilitation for your pelvic floor dysfunction? (What advantages and disadvantages do you see? Can you give me a concrete example of an event in which you felt this way?)
- Did you feel that physiotherapeutic telerehabilitation helped you achieve your goals? (If so, could you describe how? If not, what do you feel was missing; what would have been needed to achieve your goals?)
- Why were digital consultations chosen for your pelvic floor problems?
- Would you consider using telerehabilitation again in the future for treatment of pelvic floor problems? (Could you elaborate on how you feel?)
- Is there anything on your mind that we haven’t talked about?

Suggestions for general follow-up questions during the interview:

Can/could you give an example/a few examples?

Can you tell me more about it?

Can you describe it for me?

Can you elaborate on your answer?

Could you expand on your answer?

When you say ..., do you mean ...?

Do I understand you correctly when you say ...?

What did it mean to you?

## Appendix 2

### Procedure Plan

**Table:**

	What to do?	Who?	How?
1)	The entire text is read several times to get a sense of the whole.	HJ, ML, and AG	Read separately.
2)	The text is divided into meaning units based on the aim. These units are words, sentences, or paragraphs that are central to the text.	HJ, with support from ML	HJ extracts meaningful units from the texts of three participants, which are checked and discussed by ML before the work continues. ML checks again when all the text has been processed.
3)	The meaning units are condensed, which means that they are summarized without losing their message.	HJ, with support from ML	HJ condenses about one-fifth of the meaningful units, which ML reviews and discusses before proceeding. ML reviews the remaining condensed units.
4)	The condensed units of meaning are assigned codes. These codes are used as “labels” to explain the content.	HJ, with support from ML	HJ begins the coding process and receives ongoing support from ML to ensure that it is done correctly and that there are no inconsistencies in the codes.
5)	The codes are compared to identify similarities and differences, and then sorted into subcategories/categories.	HJ, ML, and AG	HJ, ML, and AG initiate the processing of the codes, with discussions held on an ongoing basis. ML oversees the methodology and ensures that the categories are consistent.
6)	Subcategories/categories are further analyzed and compared with each other and with the text as a whole. This may lead to the emergence of a main category.	HJ, ML, and AG	HJ, ML, and AG begin to process the categories, with continuous discussion. ML oversees the methodology.
	In content analysis, context is taken into account throughout the interpretation process. The transcribed text is read in its entirety throughout the analysis process.	HJ, ML, and AG	
